# Evaluation of the Objective Posturo-Locomotor-Manual Method in Patients with Parkinsonian Syndromes

**DOI:** 10.3389/fneur.2013.00095

**Published:** 2013-07-19

**Authors:** Theresa Zackrisson, Filip Bergquist, Björn Holmberg, Bo Johnels, Thorleif Thorlin

**Affiliations:** ^1^Department of Clinical Neuroscience and Rehabilitation, Institute of Neuroscience and Physiology, University of Gothenburg, Gothenburg, Sweden; ^2^Department of Pharmacology, Institute of Neuroscience and Physiology, University of Gothenburg, Gothenburg, Sweden; ^3^Department of Neurology, Sahlgrenska University Hospital, Gothenburg, Sweden; ^4^Department of Neurological Rehabilitation, Sahlgrenska University Hospital, Gothenburg, Sweden

**Keywords:** Parkinson’s disease, Parkinsonism, movement disorders, l-DOPA test, optoelectronic movement analysis, PLM test, UPDRS III

## Abstract

Objective methods for quantifying patients’ movement capacity would be useful in evaluating progression and interventions in neurodegenerative diseases. The Posturo-Locomotor-Manual (PLM) test is a standardized automated movement test developed to measure hypokinetic movements in patients with Parkinsonism. Our hypotheses were that the PLM movement time (MT) correlates with the Unified Parkinson’s disease rating scale (UPDRS III) motor section, and that the components of the PLM test correlate with the corresponding constructed domains of UPDRS III. We also evaluated the coherence between the results of the two assessment methods after a test dose of levodopa (l-DOPA). We assessed motor function using the PLM method and UPDRS III in parallel, in the absence of medication and after administration of 200 mg l-DOPA, in 73 patients with moderate to advanced Parkinsonism: 47 with Parkinson’s disease (PD), 17 with multiple system atrophy (MSA), and 9 with progressive supranuclear palsy (PSP). There was a fair correlation between the two assessment tools in the PD patients but not in the MSA or PSP patients. In the full dataset, there was a fair to good correlation between UPDRS III and the PLM MT. At group level, the UPDRS III l-DOPA test differentiated PD from MSA/PSP, whereas the PLM l-DOPA test differentiated between all three diagnoses.

## Introduction

The clinical diagnosis of Parkinson’s disease (PD) requires bradykinesia and at least one of resting tremor, rigidity, or loss of postural reflexes (1, 2). A number of different scales have been used to grade the extent of motor and non-motor symptoms and progression of PD (3). The unified Parkinson’s disease rating scale (UPDRS) is currently the most widely used and evaluated scale, and is the gold standard in clinical PD research (4, 5). It is used internationally as a clinical tool to asses Parkinsonism (6), as a marker for disease progression (7), and to evaluate the outcome of interventions (8).

As a complement to a clinical examination, objective methods have been proposed, such as gait quantification and timed tests (e.g., pronation-supination test, finger dexterity, and stand-walk-sit test) (9, 10). Appropriate symptom evaluation methods are essential, both for monitoring disease progress and for evaluating the efficacy of a growing number of treatment interventions. For motor symptoms, rater-independent quantitative evaluation methods that allow repeated measurements are desirable (11). One advantage of using objective quantitative assessment is that it reduces the elements of bias or error that can enter into an assessment process (12), such as inter-rater variability (depending upon the personal skills and experience of each examiner) and intra-rater variability (13).

Another important factor is the timing of symptom assessments after oral drug administration. The time of medication effect onset depends on the gastric emptying frequency, which may decrease with the progress of PD (14–18). In addition, some patients experience a short-lived worsening of Parkinsonian symptoms for up to 20 min after l-DOPA uptake (19). Assessing symptoms at the right time point is therefore not a trivial task. Timing is less crucial when using continuous or repeated symptom evaluation methods, as these methods record the optimal effect as well as the effect duration. Another aspect of repeated measurements is that they provide information about symptom variability, which makes it possible to decide if a medication-induced change in performance is also statistically significant in individual patients.

The Posturo-Locomotor-Manual (PLM) method was designed to quantify, in a single test, the movement aspects of postural control, locomotion, and goal-directed hand movements and the efficacy with which these movements integrate to a smooth dynamic performance (20, 21). This test is automated and rater-independent, and although it tests a limited set of movements, the movements are chosen to reflect an everyday situation and to sample capacities in several domains including balance, locomotion, and basic manual function. The PLM test has been used for more than 10 years in some Swedish neurological clinics to consecutively evaluate hypokinetic symptoms in PD patients and l-DOPA responsiveness, and it is now a commercial product. However, it has not been validated against other methods, and in particular not against UPDRS III, which is the current gold standard for assessing motor symptoms in PD (5, 22, 23).

We hypothesized that the PLM method correlates with the UPDRS III, and that the different phases (Postural, Locomotor, and Manual) of the PLM test correlate with constructed domains of the UPDRS III. We also hypothesized that a significant improvement in the PLM test after a test dose of l-DOPA predicts an improvement in UPDRS III scores.

## Materials and Methods

### Patients

We retrospectively studied clinical rating and PLM measurements in 73 patients with Parkinsonism who were referred to the movement laboratory to perform an l-DOPA test: 47 with PD, 17 with multiple system atrophy (MSA), and 9 with progressive supranuclear palsy (PSP). Patients’ characteristics are presented in Table 1.

**Table 1:** **Patient characteristics**.

Diagnosis	PD (*n* = 47)	MSA (*n* = 17)	PSP (*n* = 9)
Age (mean ± SD, range)	61.9 ± 7.2 (52–76)	53.9 ± 9.0 (43–68)	64.7 ± 10.4 (44–75)
Males/females	29/18	12/5	7/2
Hoehn and Yahr _ON_ (median, range)	2.5, 1–3		
UPDRS _OFF_ (mean ± SEM, range)	35.7 ± 1.7, 6–59	31.6 ± 3.1, 15–61	32.7 ± 2.6, 17–46
UPDRS _ON_ (mean ± SEM, range)	19.1 ± 1.7, 2–61	29.7 ± 3.1, 13–60	29.8 ± 7.0, 18–44
MT _OFF_ (mean ± SEM, range)	3.5 ± 0.4, 1.6–19.3	3.8 ± 0.6, 1.8–10.6	8.6 ± 3.9, 2.6–38.7
MT _ON_ (mean ± SEM, range)	2.1 ± 0.1, 1.2–4.5	3.6 ± 0.5, 1.7–8.7	7.9 ± 3.25, 1.8–30.8
Disease duration (mean ± SD)	13.1 ± 5.7	3.4 ± 2.1	4.0 ± 3.6
Treatment (mg LDE, mean ± SD)	1258 ± 605	492 ± 525	494 ± 578

Motor function was evaluated by the PLM method and UPDRS III in OFF and ON states to determine if the patient responded positively to a test dose of l-DOPA. The patients had given informed consent to the testing procedure before the assessments, and retrospective analysis of the anonymized collected data was approved by the Regional Ethical Review Board in Gothenburg, Sweden.

The PLM results for three PD patients with freezing of gait phenomena that yielded very long movement times (MTs) in OFF were omitted from the correlation analysis, as they were clear outliers in the PLM test and might have skewed this analysis. For seven patients, only the total UPDRS III scores were documented in the medical records; the subscores for the different items were not available. Those seven individuals were all PD patients, and were excluded from the correlation analyses between PLM and the different UPDRS III domains (data presented in Table 3).

### The PLM method

**Figure 1:**
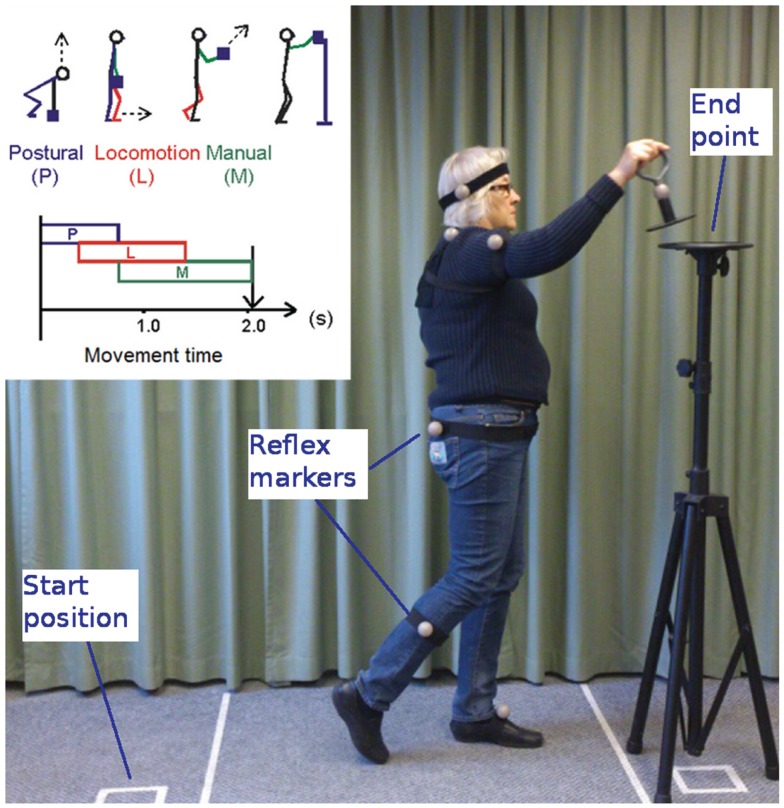
**Illustration of the PLM method**.

The PLM method is designed to assess movement patterns in patients with hypokinetic syndromes. The test movement is a compound movement involving a postural phase P (rising up), a locomotion phase L (walking), and a manual phase M (pendulous arm movement and positioning of a test object on a pedestal, Figure 1). An infrared camera system is used to register body movements by tracking the position of six reflective ball markers of 4 cm diameter. The markers are attached to the patient’s head; to the shoulder, arm, hip, and calf of the most severely disabled side of the body; and to the contralateral foot. A seventh marker is located on a test object consisting of a 500 g metal handle on a cylindrical horizontal plate (24–26). An automated tracking algorithm is used to identify the markers and analyze the recorded data. The total MT is calculated along with the duration of the different movement phases P, L, and M (27), and the software produces a report after a full test session.

At the start of the test, the patient is asked to stand erect with feet together at a clearly marked start position with the test object on the floor beside them. When instructed, the participant lifts the object from the floor, walks forward as quickly as possible, and places the object on a stand located 1.5 m away at chin height. This movement is performed three times, after which the patient rests for a short while. Three consecutive movements constitute a measurement group. Ten measurement groups are collected to allow the patient to reach a performance plateau. Mean MT, P, L, and M durations are automatically calculated from the three best consecutive groups. All nine individual measurements from these three groups are used to calculate standard deviations for each variable.

### Test procedure

The PLM test and the UPDRS rating were carried out in the same clinical movement laboratory. All antiparkinsonian medication was withheld for at least 12 h prior to performing the l-DOPA test, as recommended in published guidelines (10). A trained movement disorder physiotherapist administered the motor part of the UPDRS as described by Goetz et al. (6) before the PLM test started (UPDRS III OFF), and 69 min (±32 min) after administration of 200 mg of l-DOPA (UPDRS III ON).

**Figure 2:**
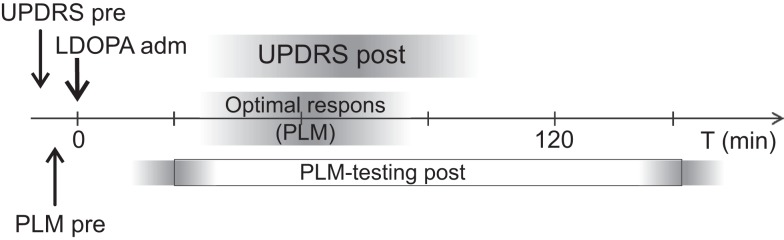
**Timeline for the l-DOPA test**.

A trained biomedical analyst instructed all patients and performed the PLM test. First, 10 baseline groups of PLM measurements were performed, and the three fastest consecutive groups were designated best mean “OFF” performance. Next, the patients were given 200 mg of l-DOPA (Madopar^®^, 200 mg) dispersed in water (28) and allowed to rest for 31 min (±20 min). Following this, two consecutive groups of PLM measurements were collected every 10 min for 2 h (Figure 2) to ensure that measurements were obtained at the time of maximum l-DOPA effect (18). The three fastest consecutive groups of measurement after l-DOPA administration were designated best mean “ON” performance; these occurred 63 ± 25 min after drug administration.

### UPDRS III domains and PLM variables

The MT variable of the PLM method was compared to the total score of the UPDRS III (UPDRS items 18–31) as well as to a version of the UPDRS III where scores for speech, facial expression, resting tremor, and postural tremor had been excluded UPDRS (−). We also analyzed the correlations between the different PLM phases and a number of corresponding constructed domains of the UPDRS III (Table 2). The UPDRS III items reflecting postural instability and gait difficulties (PIGD, items 27–30) were evaluated separately against the PLM variables MT, P, and L.

**Table 2:** **UPDRS III domains and PLM variables**.

PIGD* (postural domain + gait)	Item 27	Arising from a chair
	Item 28	Posture
	Item 29	Gait
	Item 30	Postural stability
Postural domain	Item 27	Arising from a chair
	Item 28	Posture
	Item 30	Postural stability
Rigidity	Item 22	Neck
Leg domain	Item 26	Leg agility
	Item 29	Gait
Rigidity	Item 22	Leg
Hand/arm domain**	Item 23	Finger taps
	Item 24	Opening and closing the fist
	Item 25	Pronation and supination
Rigidity**	Item 22	Arm

*Postural instability and gait difficulty score.

**Most affected side.

### Positive l-DOPA response

Patients who improved by six points or more in UPDRS III score after a test dose of 200 mg l-DOPA were considered to have a positive l-DOPA response (29). We also report the results and concordance between the two tests with some alternative cut off values for acute l-DOPA response: improvements in UPDRS III of 10 points or more, 30, and 50%. A change in MT was considered positive if the confidence interval for MT OFF (MT OFF ± 1.96 SD) was numerically higher and disjoint from the confidence interval for MT ON (MT ON ± 1.96 SD).

### Statistical methods

Mean, SD, SEM, median, and range of data were used for descriptive purposes. All correlation analysis was performed using Spearman’s non-parametric correlation coefficient. All *p*-values were two-tailed and conducted at the 5% significance level. The following criteria were used to evaluate the strength of the correlations: fair (0.25–0.49), good (0.50–0.74), and excellent (0.75 and above) (30). Agreements between UPDRS III and PLM MT l-DOPA tests were tested with McNemar’s test after categorizing the response as positive or negative. Analysis of UPDRS III and PLM results at the level of diagnostic groups was done with two-way ANOVA, with diagnosis and treatment state as independent factors and UPDRS III or PLM as the dependent variables. *Post hoc* comparisons were made with Bonferroni corrected *t*-tests.

## Results

### Unified Parkinson’s disease rating scale III

The baseline OFF UPDRS III scores and the assessments after a test dose of l-DOPA are given in Table 1.

**Figure 3:**
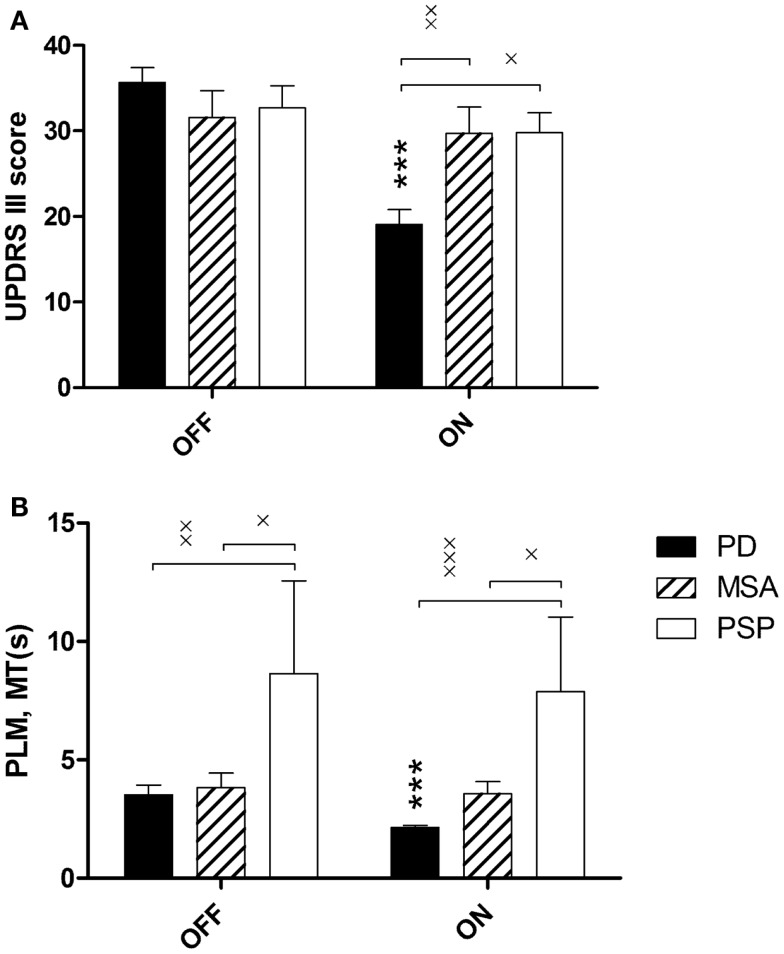
**(A)** UPDRS III scores before (OFF) and after (ON) 200 mg l-DOPA, stratified over the three diagnoses: Parkinson’s disease (PD), multiple system atrophy (MSA), and progressive supranuclear palsy (PSP). **(B)** PLM mean movement time, MT(s), duration. Main effects of diagnosis and treatment state were analyzed with repeated measure two-way ANOVA followed by Bonferroni corrected *t*-tests where ***p < 0.001, PD OFF vs. PD ON, *p < 0.05, **p < 0.01.

The effect of l-DOPA treatment was evaluated by repeated measure two-way ANOVA with diagnosis and treatment state as independent variables and UPDRS III as the dependent variable. There was a significant interaction between diagnosis and treatment state: *F*(2,70) = 17.5, *p* < 0.0001, and a significant main effect of treatment state: *F*(1,70) = 24.2, *p* < 0.001. The interaction was explained by a reduction in ON UPDRS III scores in PD patients (Figure 3A).

### Posturo-locomotor-manual

The baseline OFF PLM results and the results after a test dose of l-DOPA are given in Table 1.

The effect of l-DOPA treatment was evaluated by repeated measure two-way ANOVA using diagnosis and treatment state as independent factors. There was a significant main effect of treatment state: *F*(1,70) = 5.2, *p* = 0.0258, and of diagnosis: *F*(2,70) = 7.1, *p* = 0.0016. *Post hoc* analysis revealed that PSP patients had longer MT than the other patient groups in both ON and OFF, and that MT decreased in PD patients but not in MSA or PSP patients after l-DOPA (Figure 3B).

### Correlations between UPDRS III and the PLM test

In the full dataset, fair to good correlations were found between most UPDRS III domains and the corresponding PLM phases. There was also significant correlation between the l-DOPA induced effects observed with UPDRS III and PLM (Table 3). Overall, the correlations were lower in OFF than in ON and OFF-ON (magnitude of change after l-DOPA administration). No or low correlations were found between the L phase and leg rigidity and between the M phase and the corresponding constructed UPDRS III domains. Fair and significant correlations between UPDRS III and PLM MT were found with PD patients in OFF, ON, and OFF-ON, but not consistently for MSA and PSP patients, with the exception of OFF-ON in the small sample of PSP patients, where there was excellent correlation (Table 4).

**Table 3:** **Correlation between PLM results and the UPDRS in all patients**.

	OFF			ON			OFF-ON		
	*r*	*p*-Value	*n*	*r*	*p*-Value	*n*	*r*	*p*-Value	*n*
MT vs. UPDRS (total)	0.37	0.0017	70*	0.58	<0.0001	73	0.60	<0.0001	70*
MT vs. UPDRS (−)	0.35	0.0042	64**	0.56	<0.0001	66**	0.58	<0.0001	64**
MT vs. bradykinesia	0.39	0.0013	64	0.62	<0.0001	66			
MT vs. PIGD	0.56	<0.0001	64	0.62	<0.0001	66			
P phase vs. postural domain	0.36	0.0030	64	0.65	<0.0001	66			
P phase vs. neck rigidity	0.22	0.0806	64	0.41	0.0006	66			
P phase vs. PIGD	0.48	<0.0001	64	0.70	<0.0001	66			
L phase vs. leg domain	0.51	<0.0001	64	0.64	<0.0001	66			
L phase vs. leg rigidity	−0.16	0.2186	64	0.04	0.7474	66			
L phase vs. PIGD	0.55	<0.0001	64	0.53	<0.0001	66			
M phase vs. hand/arm domain***	0.09	0.4925	64	0.29	0.0172	66			
M phase vs. arm rigidity***	−0.07	0.5828	64	0.12	0.3310	66			

*UPDRS (−) excluding scores for speech, facial expression, resting tremor, and postural tremor*.

*PIGD postural instability gait difficulty score (UPDRS Items 27–30)*.

*The PLM results for three PD patients in OFF were omitted from the correlation analysis due to freezing of gait phenomena yielding very long movement times.

**UPDRS III subscores were not available for all patients.

***Most affected side.

**Table 4:** **Correlation between PLM MT(s) and UPDRS III for each diagnosis**.

	OFF			ON			OFF-ON		
	*r*	*p*-Value	*n*	*r*	*p*-Value	*n*	*r*	*p*-Value	*n*
PD	0.47	0.0013	44*	0.44	0.0019	47	0.47	0.0015	44*
MSA	0.49	0.0448	17	0.46	0.0635	17	0.05	0.8544	17
PSP	0.27	0.4860	9	0.22	0.5755	9	0.75	0.0210	9

*The PLM results for three PD patients in OFF were omitted from the correlation analysis due to freezing of gait phenomena yielding very long movement times.

### l-DOPA response at subject level

The majority of PD patients responded positively to a test dose of l-DOPA as measured with either method. A decrease of six or more points in UPDRS III classified 40/47 of the PD patients as responders, compared to 34/47 with the PLM method; the concordance between the two test methods was 70%. Few of the MSA patients showed improvement after medication with either method (UPDRS 4/17, PLM 3/17); here, the concordance between the two methods was 59%. In the small sample of PSP patients, about 20% responded positively to l-DOPA (UPDRS 2/9, PLM 2/9), with a concordance of 78% between the methods (Table 5).

**Table 5:** **l-DOPA responses with the two measuring tools**.

UPDRS improvement cut off	PD (*n* = 47)				MSA (*n* = 17)				PSP *n*(9)			
	≥6 *p*	≥10 *p*	≥30%	≥50%	≥6 *p*	≥10 *p*	≥30%	≥50%	≥6 *p*	≥10 *p*	≥30%	≥50%
Positive in PLM	34	34	34	34	3	3	3	3	2	2	2	2
Negative in PLM	13	13	13	13	14	14	14	14	7	7	7	7
Positive in UPDRS	40	35	34	28	4	0	1	0	2	0	0	0
Negative in UPDRS	7	12	13	19	13	17	16	17	7	9	9	9
Concordant positive in PLM (%)	64	57	55	47	0	0	0	0	11	0	0	0
Concordant negative in PLM (%)	6	11	11	15	59	82	76	82	67	78	78	78
Discordant (%)	30	32	34	38	41	18	24	18	22	22	22	22

The Parkinson’s disease patients who were discordantly l-DOPA negative in the PLM test displayed significantly larger variability in PLM performance before and after l-DOPA administration. The PLM MT standard deviation in this group was 339% of the l-DOPA induced change in performance, whereas it was 70% in the group that was l-DOPA responsive with both methods, and 29% in the group that was positive in the PLM test only [one-way ANOVA of logarithmized values: *F*(2,51) = 12.5, *p* < 0.0001]. A *post hoc t*-test revealed significant differences between PLM negative/UPDRS positive patients and the other two groups (*p* < 0.0001 in both cases).

## Discussion

This study was designed to validate the objective and quantitative PLM method for assessing Parkinsonism, by comparing this method with the commonly used clinical rating scale UPDRS part III. There was a fair correlation between the PLM (MT) and the UPDRS assessment in PD but not in MSA and PSP patients. Overall, the correlations were somewhat stronger in ON and OFF-ON than in OFF. The postural and locomotive phases of the compound PLM movement showed good correlation to the pre-constructed corresponding domains in the UPDRS when evaluated in the full dataset. However, the movement phase of the PLM test did not correlate with the hand and arm related domains of the UPDRS.

The degree of correlation between the two evaluation methods may appear modest in places, but since our purpose was to examine the relationship between a subjective clinical rating scale and a quantitative analysis of motor function, establishing a fair correlation is a clinically useful validation of the quantitative method. As the PLM method is more expensive than clinical rating, and is also time consuming if the medication response profile is followed beyond maximum effect, a very high correlation would have suggested that the PLM test adds no information beyond that obtained from the UPDRS.

There are several differences between the UPDRS III and the PLM test. In particular, the UPDRS III measures some motor features that go undetected in the PLM test, such as tremor, speech, and facial expression. The PLM test also contains no alternating movements, which may explain the lack of correlation between the arm/hand domain of the UPDRS III and the M phase in the PLM test. We hypothesized that the pendulous arm movement of the M phase might correspond better to arm rigidity than to hand bradykinesia, but this assumption was not supported by the correlation analysis.

The presence of tremor strongly influences the UPDRS III scores, but has no practical effect in PLM. Nevertheless, removing tremor and facial features from UPDRS III did not improve the correlation between UPDRS III and PLM MT. This suggests that the tremor rating does not contribute fundamentally different information from that captured by PLM. Consequently, it appears that the PLM test gives a fair to good estimate of overall Parkinson symptomatology, but it is clear that the PLM test covers fewer domains of Parkinsonism and its face validity is therefore lower than that of UPDRS (7). The advantage of the PLM test is the rater-independent outcome and the repeated measurements that eliminate inter-rater variability and provide a measure of the patient’s performance and symptom variability. As a consequence, it is possible to determine whether a treatment has a significant effect in a single patient. It is also our experience that, in some cases, being able to present objective improvements over time has a pedagogical value and may improve compliance.

We registered significant improvements in PD patients following acute l-DOPA treatment with both the UPDRS III and the PLM method, but no such improvements were seen in the MSA or PSP group. Interestingly, the PLM test and the UPDRS III provided different information on a group level, as PSP patients had significantly higher MT than the other diagnostic groups but were not singled out by their UPDRS III scores. However, the PSP group was small, and the significantly higher MT needs to be confirmed in a larger population with atypical Parkinsonism.

Different cut off values for UPDRS improvements have been used to categorize subjects as l-DOPA responsive or not. Schrag et al. (29) suggested that a decrease of more than five points in UPDRS III after l-DOPA administration represents a minimal clinically relevant improvement in motor ability. Others have defined responders as those improving by 30% in UPDRS III score (31); however, the outcome then largely depends on baseline score, so more advanced patients have lesser probability of demonstrating a positive effect. Because a clinically relevant improvement is the most appropriate reason for introducing or continuing treatment, we argue that a six-point cut off is preferable. This is further corroborated by our finding that the 6-point cut off identified more l-DOPA responders than a 10-point, 30, or 50% improvement in UPDRS III, and had the best overlap with the PLM method (Table 5).

Because the PLM test covers a smaller subset of l-DOPA responsive features, the somewhat lower ratio of l-DOPA responders revealed by the PLM method in our material was as expected. However, it was also evident that the group of patients who were l-DOPA-responsive with UPDRS III, but not PLM, displayed significantly larger variability in PLM MT both in OFF and ON, whereas patients who were positive only with the PLM test had a significantly lower variability in MT_ON_. Patients who were congruent in both tests showed low variability both in OFF and ON. This was an unexpected but interesting finding, indicating that the PLM method has the ability to detect variability in motor performances both OFF and ON medication, and that the variability differs between patients; some patients have a large variability in OFF, others have a large variability in both OFF and ON, and yet others have a very small variability after administration of l-DOPA as compared to before administration of l-DOPA. This may to some extent explain why the PLM test is less sensitive than UPDRS III in detecting l-DOPA improvement, because for a positive l-DOPA response in the PLM test the improvement has to be statistically significant, but with UPDRS it only has to be more than five points.

This raises the question of the best timing of the assessment after an l-DOPA dose. The repeated measurements obtained with the PLM method reveal highly variable performances in some patients. The variability in performance will not be detected if a single UPDRS rating is performed before and at a defined time point after treatment (15, 18, 32, 33). This may be an advantage of the PLM test, but the clinical relevance of detecting motor performance variability is to our knowledge not known. The repeated PLM tests indicated that optimal improvement after l-DOPA occurred 63 ± 25 min after administration, so a 1-h wait after administering l-DOPA dispersed in water before evaluation of effect appears appropriate. All UPDRS III ratings ON medication in the present study were performed within the optimal time span.

One obvious problem with the PLM test is that patients who cannot walk cannot perform the test. Also, patients who experience freezing of gait phenomena may produce disproportionately long MTs. Although this does not preclude assessment of l-DOPA response in patients who can walk after the test dose, it introduces a non-linear component to the PLM test. In the present dataset, we excluded 3 out of 73 patients due to obvious freezing of gait in OFF. Most of these patients could still be assessed in ON, and the exclusion was made only because they were outliers in the correlation analysis. Despite this general limitation, the PLM test may still be useful for evaluating whether freezing of gait is l-DOPA responsive in individual patients.

The current study population had relatively long mean disease duration, and had passed the early stages when correct diagnosis is more difficult. A prospective study of patients who have recently presented with their first signs of hypokinetic movement disorders would be needed to determine whether the PLM test can provide any additional diagnostic information that might aid the clinician.

## Conclusion

We found a fair correlation between the PLM test and a simultaneous UPDRS III rating in PD patients OFF and ON medication, despite the fact that the PLM test only samples some of the disabilities rated with the UPDRS III. On a group level, the PLM method distinguished between PD, MSA, and PSP patients. The PD patients could be identified by a positive response to l-DOPA, and the PSP group was significantly slower than both MSA and PD patients. The PLM method provides a measure of motor performance variability ON and OFF medication that cannot easily be obtained with UPDRS III. Our findings suggest that the PLM test is a valid method for assessing motor performance in OFF and ON state, as well as l-DOPA responsiveness in patients with Parkinsonism at moderately advanced stages. It remains to be seen whether the PLM test can also reliably detect positive l-DOPA responses in *de novo* PD patients less marked by their disease.

## Conflict of Interest Statement

The research was conducted in the absence of any commercial or financial relationships that could be constructed as a potential conflict of interest.
